# Supplementary material for: Identification and Functional Analysis of the Caffeic Acid O-Methyltransferase (COMT) Gene Family in Rice (Oryza sativa L.)
Source: Int J Mol Sci. 2022 Jul 31;23(15):8491. doi: 10.3390/ijms23158491 (PMC9369235; doi:10.3390/ijms23158491)
Supplement: Supplementary file 1 [file ijms-23-08491-s001.zip › Figure S1.pdf]

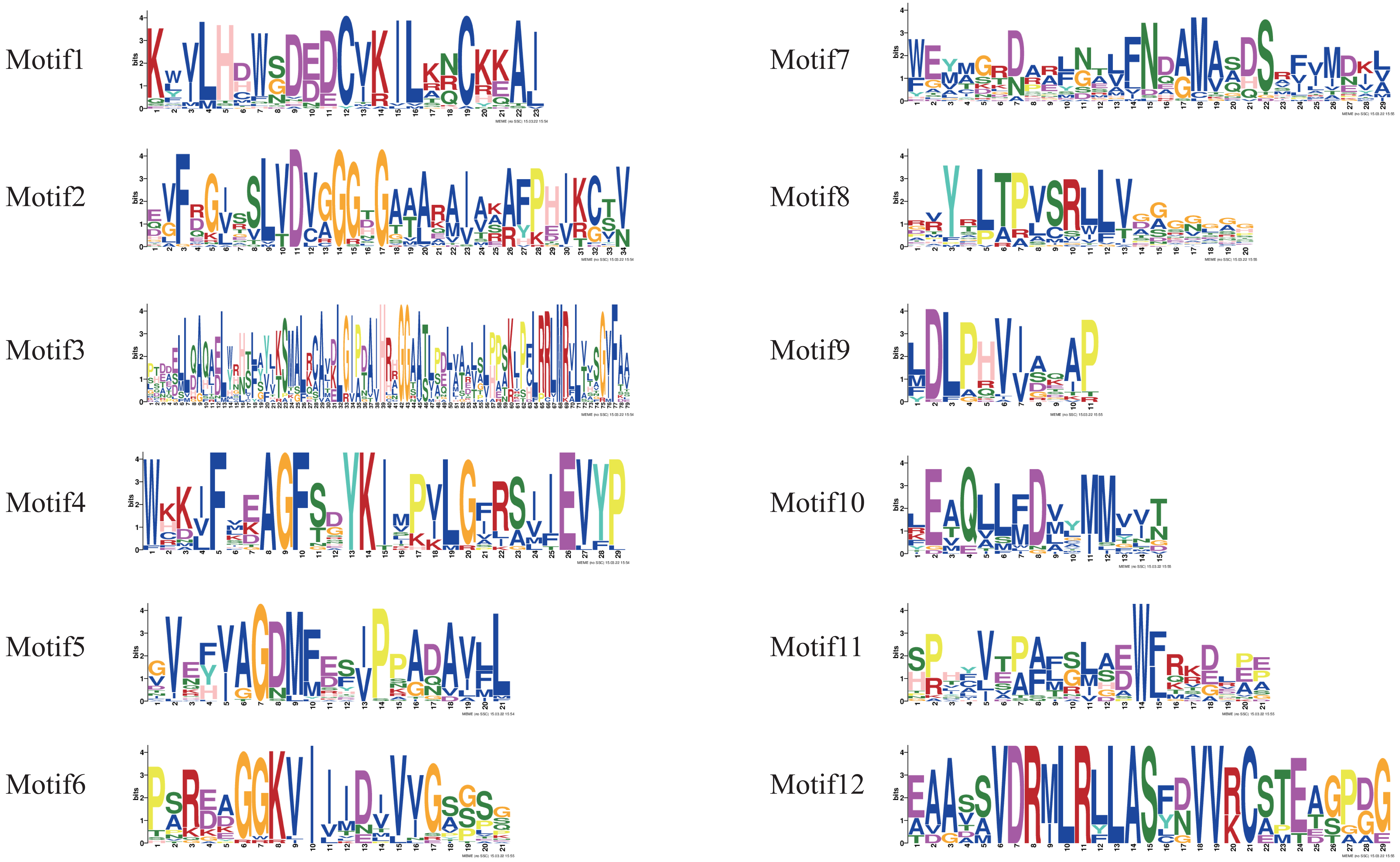


Figure S1. 12 motifs of *OsCOMTs*
